# Climate change and health: translating the call of the hummingbird

**DOI:** 10.1016/j.ebiom.2023.104718

**Published:** 2023-07-12

**Authors:** eBioMedicine

“We are hurtling towards disaster, eyes wide open. It is time to wake up and step up.”

António Guterres, UN Secretary General, June, 2023

In this address to journalists, Guterres was mainly calling out the fossil fuel industry and its enablers, but we can and should all feel concerned and compelled to act. During the past few years, no one has been spared the striking manifestations of climate change across the world, including extreme heat waves, historic droughts, floodings, landslides, or substantial forest fires and their far-reaching smoke clouds. The effects of these manifestations on human health are well documented and the statistics shared every year by organisations such as the Intergovernmental Panel on Climate Change or The Lancet's Countdown on Health and Climate Change reveal the sobering and dire truth of our world. To feel overwhelmed is a natural reaction, but we need to remember that we have the power to act. We should all be like the hummingbird in the famous folktale who, instead of being transfixed by the fire devastating his forest as the rest of the animals are, decides to do what he can and starts carrying drops of water in his small beak to the fire, despite his efforts potentially appearing small and in vain. We should all be thinking about what we can do to limit the climate change crisis.

At *eBioMedicine*, we started to think about how to act against climate change, as editors, about 2 years ago. At that time, several groups of researchers and funding agencies (eg, the HERA group in Europe, the US National Science Foundation, and the US National Institutes of Health) shared their suggested agendas for future research into the ways in which climate change affects human health. They highlighted gaps in the understanding of the biological mechanisms of these interactions. Therefore, use of our translational science journal to help identify and fill these knowledge gaps seemed like a natural process. We have gathered a collection of Reviews, Articles, Correspondences, and Personal Views that we proudly present as a Series for the July, 2023, issue.

The effect of air quality on human health is one of the most studied effects of climate change. However, Li-Zi Lin and colleagues from Sun Yat-sen University in China noticed that its influence on infants younger than 12 months was less documented. Therefore, in a narrative review, they presented the associations between air pollutants and various infant outcomes, such as adverse birth outcomes, infant morbidity and mortality, respiratory health, early neurodevelopment, and early growth. Several associations were identified; some were indirect, through the health of the birthing parent or the exposure period during pregnancy, and some were direct, through effects on developing organs. The patterns of the associations varied by type of pollutants, exposure periods, and lengths of exposure. Although these associations are clear and support a call for action to further reduce ambient air pollution, the authors recommended broadening research to study the effects of ambient air pollutants other than the commonly investigated particulate matter (PM) and nitrogen dioxide. In a personal view, Ruzmyn Vilcassim and George D Thurston from the University of Alabama and New York University in the USA highlighted additional methodological issues hampering the proper assessment of air-pollution exposure in research studies. They recommended, in particular, considering not only particle mass concentrations but characteristics and deploying low-cost sensors for extensive and personalised monitoring especially in low-income and middle-income countries**.**

Carbon monoxide and PM are the main constituents of air pollution that comes to mind, but Chris C Lim and colleagues from the University of Arizona in the USA reminded us that harmful algal blooms can release toxins that can be incorporated into aerosols and transported inland, representing another source of pollution. Although aerosols from a few species were associated with respiratory outcomes, substantial uncertainties remain regarding the effects of long-term exposure, the effects on susceptible subpopulations, and the effects of concurrent exposures with other toxins or aerosol sources. We have also published a few original research articles furthering our understanding of the health effects of air pollution. For example, Lisa Miyashita and colleagues from Queen Mary University of London, UK, studied the effects of PM_10_ (ie, PM smaller than 10 μm) from the London Underground transport system in vitro, in human cells, and in mice. They observed that PM_10_ increased the susceptibility to pneumococcal infection by increasing bacterial adhesion and entry in airway epithelial cells. Jonathan Allouche and colleagues from Université Côte d'Azur in France, took advantage of the COVID-19 lockdown to study the large-scale effects of variations in air pollutants in a real-world setting. In their single-centre, matched-cohort study, they observed a weakening of the antiviral cellular response (represented by IFNγ production) correlated with an increase in pollutant exposures.

If the immune response to infectious diseases could be altered by air pollution, the dynamics of these infections are themselves affected by climate change. For example, global warming has been identified as a risk factor for increased dengue virus transmission in various studies. To provide public health policy makers with more locally useful evidence, Yohannes Tefera Damtew and colleagues from the University of Adelaide in Australia, conducted a systematic review and meta-analysis of 54 studies to assess the effects of high temperatures and heat waves on dengue incidence across different climate zones. Their review substantiated that high temperatures increase the risk of infection, but this risk varies across climate zones. Although heat-wave events were also associated with increased risk of infection, the strength of evidence was considered limited by the authors. Future studies should focus on further exploring these associations at a regional level. Another category of climate-sensitive diseases are viral respiratory infections. To prevent future crises, such as the COVID-19 pandemic, understanding the mechanisms underlying the emergence and transmission of such infections is key. However, as highlighted in the Review by Yucong He and colleagues from Tsinghua University in China, epidemiological studies have not yet been able to provide real-life support of the biological mechanisms identified in vitro or in animal models. This lack of support can be explained partly by the complexity of the associations and the interplay between biological, socioeconomic, and ecological factors and evolving human–animal–environmental interfaces in real life. Transdisciplinary research involving epidemiologists, biologists, and climate scientists is therefore essential. This conclusion can be generalised beyond infectious diseases to all areas affected by climate change and influencing health. How climate change will affect crop yields and nutrients in these crops and how these crops will modify health of the population is another example of the importance of collaborative research efforts.

Multidisciplinary research is also needed to understand how climate change leads to increases in allergic respiratory diseases. As increased temperatures and air pollutants modify the sources of airborne allergens (ie, in terms of seasonality, nature, and amounts), which affect the health of people who are allergic, experts in epidemiology, aerobiology, meteorology, and immunology can provide further understanding into this interaction. Paul J Beggs and colleagues are such experts, who teamed up to write a Review describing current knowledge on airborne allergens and explored three translational mitigation approaches to improve the health outcomes of affected populations. The proposed approaches rely on technological advances to reduce exposure to allergens by ensuring automated, real-time monitoring of allergens; creating reliable allergen-forecasting models; and sharing their results and additional information to the population. As well as these promising approaches, the Review also clarified that further research is needed; current studies have been mostly conducted in high-income countries and on a few key species. Furthermore, literature on the effects of climate change on respiratory diseases due to allergies is scarce.

As the effects of climate change are becoming more noticeable, investigating adaptation and mitigation strategies is of the utmost importance. Shehla Zaidi from Aga Khan University in Pakistan proposed an application of this message to overcome increasing health shocks due to floods in her country. On a global scale, Hayon Michelle Choi and colleagues from the Multi-Country Multi-City Collaborative Research Network explored how green space modifies the relationship between heat and mortality. Via data from 452 locations across 24 countries, cities with more green spaces had lower heat-mortality risk than cities with fewer green spaces, supporting findings from smaller studies. Although this large-scale study provides clear evidence of the benefits of green spaces, the authors emphasise the importance of further research at a much smaller scale (eg, at the neighbourhood level) to identify susceptible populations and inform policy makers on the most effective interventions to reduce health inequalities.

Climate and human health are clearly linked, but climate is only one aspect of planetary systems that affect our wellbeing. In a Nature article published in May 2023, Johan Rockström and colleagues proposed a model of eight safe and just Earth-system boundaries that quantify the state of climate, the biosphere (ie, the natural ecosystem area and the functional integrity of all ecosystems), water (ie, surface and groundwater) and nutrient cycles (nitrogen and phosphorus), and aerosols. They observed that seven of these boundaries were already exceeded. Furthermore, Linn Persson and colleagues from Stockholm Environment Institute, Sweden, quantified the planetary boundary for novel chemical entities and concluded that this boundary was exceeded as annual production and releases increase at a faster rate than the global capacity for assessment and monitoring. Production and releases should urgently be reduced and research into existing entities is needed to anticipate and, hopefully, mitigate their risks. In an attempt to fill existing knowledge gaps, *eBioMedicine* published some Articles on this topic. In all samples of a population-based cohort study of 738 people who were pregnant and their children, Astrid Sevelsted and colleagues from the University of Copenhagen, Denmark, detected perfluoroalkyl substances (ie, PFOS and PFOA, which are persistent and bio-accumulative chemicals). Higher concentrations of these substances were associated with reduced intrauterine growth. Interestingly, the two substances had opposite effects on height and concentration of PFOS interacted with the sex of the child (ie, 6-year BMI was lower in female children but higher in male children with increasing concentrations), suggesting a potential mechanism of action. In a proof-of-concept case series, Thomas Horvatits and colleagues from the University of Hamburg, Germany, observed an accumulation of microplastics in the livers of people with cirrhosis but not in the livers of healthy people. Further research is needed to understand this observation and understand whether this accumulation is a cause or a consequence of tissue fibrosis.

To prepare our Series, we had the opportunity to meet and discuss with researchers and clinicians. One thing we noticed was that, for individuals interested in topics related to climate change and health, research often represents only a part of their efforts. Thus, we proposed sharing inspirational examples in a group of Correspondences. Four contributions from medical students from Medical Students for a Sustainable Future inaugurate the Series. Their lived experiences, the actions they took to try to reform the systems that are responsible for the degradation of the environment, the effects on the health of their patients, and the results they have already achieved (eg, the integration of climate health into the medical curriculum in their universities) should be a reflection point and call for action. For example, an individual could organise a panel session on their specialty at a conference and invite their community to reflect on how climate change affects their field, conduct an audit in their laboratory or department to reduce waste and energy consumption, or get involved in advocating at agencies for specific funding for multidisciplinary research that has been historically underfunded.

Our aim for the Series on climate change and health is to encourage hope in the definition of Jane Goodall, not “passive wishful thinking” but “real hope, which requires action and engagement”. This quote opened our December, 2021, Editorial. Although meaningful research has been published since, the Reviews presented in our Series clarify that research is still needed, especially multidisciplinary research investigating mechanisms and mitigation approaches applied in low-income and middle-income countries or in susceptible populations. *eBioMedicine* is committed to publishing such research and welcomes research Articles, Reviews, Comments, and Correspondences on this incredibly important topic.

